# Clinical and Molecular Epidemiology of Invasive Group B *Streptococcus* Disease among Infants, China

**DOI:** 10.3201/eid2511.181647

**Published:** 2019-11

**Authors:** Wenjing Ji, Haiying Liu, Shabir A. Madhi, Marianne Cunnington, Zilu Zhang, Ziyaad Dangor, Haijian Zhou, Xiaoping Mu, Zhengjiang Jin, Aimin Wang, Xiaosong Qin, Chunyan Gao, Yuning Zhu, Xiaodan Feng, Shangyang She, Shuhua Yang, Jing Liu, Jine Lei, Lan Jiang, Zeshi Liu, Gang Li, Qiuhong Li, Qiulian Deng, Kankan Gao, Yu Fang

**Affiliations:** Xi’an Jiaotong University, Xi’an, China (W. Ji, Y. Fang);; Guangzhou Medical University, Guangzhou, China (H. Liu, Q. Deng, K. Gao);; University of the Witwatersrand, Johannesburg, South Africa (S.A. Madhi, Z. Dangor);; GlaxoSmithKline Plc, London, UK (M. Cunnington);; Harvard Medical School and Harvard Pilgrim Health Care Institute, Boston, Massachusetts, USA (Z. Zhang);; Chinese Center for Disease Control and Prevention, Beijing, China (H. Zhou);; Guangzhou Medical University, Guangzhou (X. Mu);; Hubei Maternal and Child Health Hospital, Wuhan, China (Z. Jin);; Children’s Hospital of Fudan University, Shanghai, China (A. Wang);; China Medical University, Shenyang, China (X. Qin);; Tangshan Maternal and Child Health Care Hospital, Tangshan, China (C. Gao);; Zhejiang University, Hangzhou, China (Y. Zhu);; Nanjing Maternity and Child Health Care Hospital, Nanjing, China (X. Feng);; Maternal and Child Health Hospital of Guangxi Zhuang Autonomous Region, Nanjing (S. She);; Tianjin Central Hospital of Gynecology Obstetrics, Tianjin, China (S. Yang);; Tsinghua University Hospital, Beijing (J. Liu);; The First Affiliated Hospital of Xi’an Jiaotong University, Xi’an (J. Lei);; Maternal and Child Health Care Hospital of Uygur Autonomous Region, Urumqi, China (L. Jiang);; The Second Affiliated Hospital of Xi’an Jiaotong University, Xi’an (Z. Liu);; General Hospital of Ningxia Medical University, Yinchuan, China (G. Li);; Chongqing Health Center for Women and Children, Chongqing, China (Q. Li)

**Keywords:** Group B Streptococcus, epidemiology, incidence, serotype, sequence type, clonal complex, bacteria, China, streptococci, infants

## Abstract

Invasive group B *Streptococcus* (GBS) remains a leading cause of illness and death among infants globally. We conducted prospective and retrospective laboratory-based surveillance of GBS-positive cultures from infants <3 months of age in 18 hospitals across China during January 1, 2015–December 31, 2017. The overall incidence of GBS was 0.31 (95% CI 0.27–0.36) cases/1,000 live births; incidence was 0–0.76 cases/1,000 live births across participating hospitals. The case-fatality rate was 2.3%. We estimated 13,604 cases of GBS and 1,142 GBS–associated deaths in infants <90 days of age annually in China. GBS isolates were most commonly serotype III (61.5%) and clonal complex 17 (40.6%). Enhanced active surveillance and implementation of preventive strategies, such as maternal GBS vaccination, warrants further investigation in China to help prevent these infections.

One aim of the United Nations Children’s Fund Sustainable Development Goals is to end preventable deaths among newborns and children <5 years of age by 2030 (*1*). Invasive group B *Streptococcus* (GBS), the gram-positive *Streptococcus agalactiae *bacterium, is a leading cause of illness and death among infants, including those in high-income countries. After a series of systematic reviews and a meta-analysis, a compartmental-model simulation estimated ≈319,000 GBS cases, including ≈90,000 deaths, worldwide in 2015 (*2*). Furthermore, conservative estimates for GBS-associated stillbirths were 57,000, and 33,000 invasive disease episodes occurred in pregnant and peripartum women (*2*). The overall global incidence of invasive GBS disease among infants <3 months of age in 2015 was estimated to be 0.49 (95% CI 0.43–0.56) cases/1,000 live births; the case-fatality rate was estimated at 8.4% (95% CI 6.6%–10.2%) (*3*). Two studies from China estimated the GBS incidence rate was 0.18–0.32 cases/1,000 live births (*3*). Nevertheless, China had the second highest absolute number of GBS cases among infants globally with 25,000 (uncertainty range 0–59,000) (*2*).

In 2015, China had 12.4% (17.8 million) of the 143 million global births. Systematic reviews noted the paucity of data on GBS from Asia, including China, as a major data gap (*2*–*6*), highlighting the need for prospective population-based studies. A previous study by our group reported a GBS incidence rate of 0.55 (95% CI 0.44–0.69) cases/1,000 live births in 3 hospitals in the Guangdong Province of southern China during 2011–2014 (*7*). To improve on the generalizability of our earlier study, we undertook a multicenter population-based study at 18 sentinel hospitals across 16 provinces in China. The objectives of our study were defining the epidemiology of invasive group B streptococcal disease in infants <3 months of age in China and evaluating the molecular epidemiology of invasive disease strains by serotyping and multilocus sequence typing (MLST).

## Methods

### Study Design and Population

We conducted a prospective population- and laboratory-based surveillance study for GBS in 18 urban tertiary hospitals located in 16 provinces of China during May 5, 2016–December 31, 2017. We defined cases for this study as illness among infants <3 months of age with GBS isolated from a normally sterile site, including blood, cerebrospinal fluid, soft tissues, or peritoneal or pleural fluids. We classified GBS cases as early-onset disease (EOD) for cases occurring within 0–6 days of birth and late-onset disease (LOD) for cases occurring within 7–90 days of birth (*3*). We provided sentinel hospitals with a clinical protocol to identify GBS cases. Attending physicians assessed patients to make the clinical diagnosis of invasive group B streptococcal disease. According to our protocol, blood cultures were taken before antimicrobial drug therapy for infants with clinical symptoms or signs of suspected sepsis, including but not limited to fever, breathing problems, heart rate or blood pressure abnormalities, reduced movement, fussiness, cyanosis, seizures, or limpness or stiffness. Upon laboratory confirmation of GBS culture from >1 normally sterile site, the investigator contacted the parents or guardians for consent for inclusion in the study. We acquired clinical data from the hospital information system of each site.

We also conducted a retrospective study to identify GBS cases for January 1, 2015–May 4, 2016, by using laboratory-based passive surveillance. We searched electronic information systems in laboratories for reports of GBS isolated from a normally sterile specimen. We counted infants only once, regardless of the number of positive specimens. We collected GBS isolates stored in local sites and abstracted clinical data of cases from the hospital information system.

To obtain a representative sample of China, we conducted our study in sentinel hospitals from each region of China: northeast, north, west, east, central, and south. Assuming 550,000 live births from selected hospitals during the 3-year study period and an incidence rate of 0.25 cases/1,000 live births, we expected to see 137 GBS cases in sentinel hospitals and >130 GBS cases born outside of study hospitals but seeking care in a study hospital, for a total sample size >267. 

Participating hospitals met the following inclusion criteria (*8*): large, urban, tertiary-care center; adequate research capabilities and facilities to conduct the study, including laboratory facilities and the ability to identify, process, and store GBS isolates; investigators willing to devote time to the study; and location, to ensure >1 hospital from each region. Trained site investigators in each participating hospital collected clinical data by using a standardized case report form. To estimate incidence rates by site, we obtained data on the number of live births from the information department of each participating hospital.

### Ethics Approvals

The Medical Ethics Committee of Guangzhou Women and Children’s Medical Center, Guangzhou, China, served as the central institutional review board for all facilities and approved this study (approval no. 2016050405). Each participating hospital had the option of using this approval or obtaining approval at their institution. For the prospective component of the study, we obtained written informed consent from parents or guardians of infants with invasive GBS disease. For the retrospective study, the review board waived the need for informed consent. We registered this study in the US National Library of Medicine clinical trials database (http://clinicaltrials.gov) on June 13, 2016, under registration no. NCT02812576.

### Laboratory Methods

Each local hospital laboratory performed GBS isolation, cultivation, and identification by using the following protocol. Sterile samples were inoculated in French (bioMérieux, https://www.biomerieux.com) or BACTEC (Becton Dickinson, https://www.bd.com) culture bottles and analyzed with VITEK 2 COMPACT (bioMérieux) or BD Phoenix 100 (Becton Dickinson). GBS strains were grown at 37°C in 5%–10% CO_2_ in trypticase soy agar supplemented with 5% sheep’s blood for 18–24 h, according to the manufacturer’s instructions. All GBS isolates were stored at –70°C and shipped on dry ice in standardized skim milk–tryptone-glucose-glycerol storage medium to the laboratory of Guangzhou Women and Children’s Medical Center, which is certified by Joint Commission International (https://www.jointcommission.org), for further analysis.

### Molecular Subtyping

We used multiplex PCR for Lancefield serotyping on all isolates (*9*), and tested 20% of randomly selected isolates by Strep-B-Latex (Statens Serum Institute, https://en.ssi.dk) rapid latex agglutination test kit to cross-check the PCR results (*9*,*10*). PCR assays included probes for serotypes Ia, Ib, II, III, IV, V, VI, VII, VIII, and nontypeable. We performed MLST by amplifying and sequencing the internal fragments of 7 housekeeping genes, *adhP*,* pheS*,* atr*,* glnA*,* sdhA*,* glcK*, and *tkt*. We assigned an allele number to each fragment according to its sequence, then assigned each isolate a sequence type (ST) according to the allelic profile of the 7 amplicons. We compared alleles and STs of all GBS isolates with those in the *S. agalactiae* MLST database (http://pubmlst.org/sagalactiae). We assigned GBS isolates to the same group when they shared identical alleles at 6 of the 7 loci with >1 other member of the group. We also assigned isolates to different clonal complexes (CCs).

### Data Collection and Statistical Analysis

We calculated the incidence rate by dividing the number of confirmed GBS cases in infants born in the participating hospitals by the number of live births in that hospital during the study period. To calculate the case-fatality rate, we divided the number of deaths among GBS cases by the total number of enrolled cases in each hospital.

### Sensitivity Analysis

We used the estimated average incidence from this study to calculate the expected number of cases based on the national number of births in China in 2016. We also calculated the expected number of GBS cases at each site by using the highest incidence rate of each site to adjust for possible underestimates. We also performed sensitivity analysis by using the global case-fatality rate of 8.4% (*3*) to estimate the number deaths due to invasive group B streptococcal disease in infants <90 days of age in China.

We calculated 95% CI by using the Wilson interval method and analyzed categorical data by using χ^2 ^or Fisher exact test. We evaluated incidence trends by using Cochran-Armitage trend test and considered p<0.05 statistically significant. We performed all analyses by using SAS version 9.4 (SAS Institute, https://www.sas.com) and used BioNumerics 5.1 (Applied Maths, http://www.applied-maths.com) to create minimum spanning trees.

## Results

For the full study period, January 1, 2015–December 31, 2017, we identified 304 cases of invasive group B streptococcal disease in infants from 18 hospitals, 146 (48.0%) EOD and 158 (52.0%) LOD; 123 (64 EOD and 59 LOD) were identified during the retrospective study and 181 (82 EOD and 99 LOD) during the prospective study. Of the 146 EOD cases, 82 (56.2%) were identified <24 hours after birth (Table 1; Appendix Table 1). The median length of hospitalization was 16 (interquartile range 12–26) days; infants with LOD spent more days in the hospital than did those with EOD (Table 1). Of 304 infants with GBS, 7 (2.3%) died, including 3 (3.4%) of 87 with meningitis (Appendix Table 3). Case-fatality rates were similar in the prospective (2.4%) and retrospective periods (2.2%). Attending physicians reported that 17 (5.6%) case-patients exhibited neuropathy at discharge.

**Table 1:** Characteristics of infants <3 months of age with invasive GBS disease, by disease onset, China, January 1, 2015–December 31, 2017*

Characteristics	Early-onset disease, n = 146	Late-onset disease, n = 158	Total, n = 304
Median age at diagnosis, mo (IQR)	0 (0–1)	21(15–46)	8 (0–22)
Sex			
M	74 (50.7)	64 (40.5)	138 (45.4)
F	72 (49.3)	94 (59.5)	166 (54.6)
Birth weight, g			
Median (IQR)	3,175 (2,700–3,500)	3,185 (2,800–3,500)	3,185 (2,725–3,500)
1,500–2,500	26 (17.8)	21(13.3)	47 15.5)
<1,500	5 (3.4)	8 (5.1)	13 (4.3)
Site of delivery			
Sentinel hospital	115 (78.8)	84 (53.2)	199 (65.5)
Other hospital	31 (21.2)	74 (46.8)	105 (34.5)
Median gestational age, wk (IQR)	39 (36–40)	39 (37–40)	39 (37–40)
<34	17 (11.6)	13 (8.2)	30 (9.9)
34–37	22 (15.1)	19 (12.0)	41 (13.5)
≥37	107 (73.3)	126 (79.8)	233 (76.6)
Delivery type			
Vaginal	97 (66.4)	94 (59.5)	191 (62.8)
C-section	40 (27.4)	62 (39.2)	102 (33.6)
Forceps	7 (4.8)	1 (0.6)	8 (2.6)
Unknown	2 (1.4)	1 (0.6)	3 (1.0)
Specimen type			
Blood	107 (73.3)	78 (49.4)	185 (60.9)
Cerebrospinal fluid	5 (3.4)	19 (12.0)	24 (7.9)
Blood and cerebrospinal fluid	34 (23.3)	61 (38.6)	95 (31.2)
Bacterial infections†			
Sepsis	122 (83.6)	121 (76.6)	243 (79.9)
Pneumonia‡	76 (52.1)	50 (31.7)	126 (41.4)
Meningitis	18 (12.3)	69 (43.7)	87 (28.6)
Sepsis and pneumonia	62 (42.5)	35 (22.2)	97 (31.9)
Sepsis and meningitis	7 (4.8)	43 (27.2)	50 (16.4)
Pneumonia and meningitis	2 (1.4)	2 (1.3)	4 (1.3)
Clinical symptoms†			
Fever	38 (26.0)	121 (76.6)	159 (52.3)
Breathing problems	83 (56.9)	38 (24.1)	121 (39.8)
Cyanosis	32 (21.9)	10 (6.3)	42 (13.8)
Seizures	4 (2.7)	20 (12.7)	24 (7.9)
Limpness or stiffness	5 (3.4)	10 (6.3)	15 (4.9)
Poor feeding	13 (8.9)	27 (17.1)	40 (13.2)
Irritability	4 (2.7)	15 (9.5)	19 (6.3)
Median length of hospitalization, d (IQR)	15 (12–19)	18 (13–32)	16 (12–26)
Discharge outcome			
Recovered	105 (71.9)	100 (63.3)	205 (67.4)
Transferred to other hospitals	4 (2.7)	13 (8.2)	17 (5.6)
Died	4 (2.7)	3 (1.9)	7 (2.3)
Abnormal neurology at discharge	5 (3.4)	12 (7.6)	17 (5.6)
Condition improved	12 (8.2)	16 (10.1)	28 (9.2)
Discharge requested	16 (11.0)	14 (8.9)	30 (9.9)

*Values are no. (%) except as indicated. GBS, group B *Streptococcus*; IQR, interquartile range. †Cases could have >1.  ‡As assessed by attending physician by using criteria from Zhu Futang Practice of Pediatrics (*11*).

Most (83%) case-patients, received an initial combination therapy of 2 antimicrobial drugs, including 54.2% who received empiric treatment with third-generation cephalosporins. *S. agalactiae *was 100% susceptible to cephalosporins, ampicillin, vancomycin, meropenem, and linezolid. We observed high prevalence of reduced susceptibility to tetracycline (80.1%), erythromycin (78.3%), and clindamycin (68.2%).

### Invasive GBS Disease Incidence Rates

**Figure 1:**
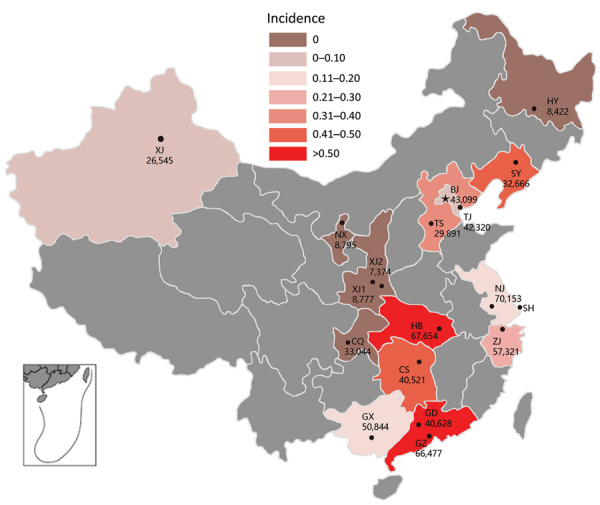
Incidence rate (cases/1,000 live births) of invasive group B streptococcal disease among infants <3 months of age by province, China. Number of live births per participating hospital is provided. Gray shaded areas did not participate in this study. Inset shows South China Sea Islands. BJ, Tsinghua University Hospital; CQ, Chongqing Health Center for Women and Children; CS, Changsha Hospital for Maternal and Child Health; GD, Guangdong Women and Children’s Hospital; GX, The Maternal and Child Health Hospital of Guangxi Zhuang Autonomous Region; GZ, Guangzhou Women and Children’s Medical Center; HB, Hubei Maternal and Child Health Hospital; HY, The First Affiliated Hospital, Harbin Medical University; NJ, Nanjing Maternity and Child Health Care Hospital; NX, General Hospital of Ningxia Medical University; SH, Children’s Hospital of Fudan University; SY, Shengjing Hospital, China Medical University; TJ, Tianjin Central Hospital of Gynecology Obstetrics; TS, Tangshan Maternal and Child Health Care Hospital; XJ, Maternal and Child Health Care Hospital of Uygur Autonomous Region; XJ1, The First Affiliated Hospital of Xi’an Jiaotong University; XJ2, The Second Affiliated Hospital of Xi’an Jiaotong University; ZJ, Women’s Hospital, Zhejiang University.

**Figure 2:**
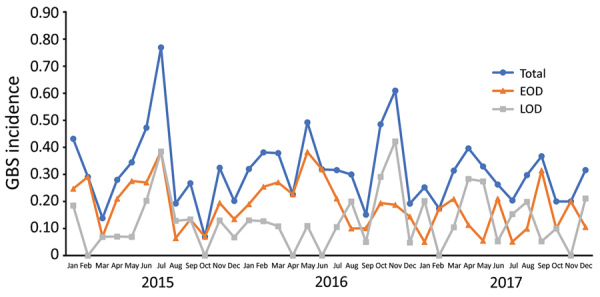
Incidence rate of invasive GBS disease (no. cases/1,000 live births) among infants <3 months of age in participating regions of China, 2015–2017. EOD, early-onset disease; GBS, invasive group B *Streptococcus*; LOD, late-onset disease.

Of 304 GBS cases, 199 occurred in infants born at sentinel hospitals, 115 EOD and 84 LOD cases. Sentinel hospitals reported 634,531 live births during the study period, 388,005 during the prospective period and 246,526 during the retrospective period (Table 2; Figure 1). The calculated overall incidence of invasive GBS disease was 0.31 (95% CI 0.27–0.36) cases/1,000 live births, and incidence was similar between the prospective (0.31 cases/1,000 live births) and retrospective periods (0.32 cases/1,000 live births) (Appendix Table 2). The incidence of EOD was 0.18 (95% CI 0.15–0.22) cases/1,000 live births and LOD was 0.13 (95% CI 0.11–0.16) cases/1,000 live births. We did not see seasonal variation in incidence rates (Figure 2).

**Table 2:** Live births and incidence of invasive GBS disease among infants <3 months of age, by region and sentinel hospital, China, January 1, 2015–December 31, 2017*

Region	Births	Case-patients born in sentinel hospitals	Expected cases/ region†	Hospital‡	Live births								Expected cases/ hospital†
										GBS case-patients born			
						GBS cases				in study hospitals			
						Total	EOD	LOD		Total	EOD	LOD	
NE	41,088	16 (0.39)	31	SY§	32,666	34	16	18		16 (0.49)	11 (0.34)	5 (0.15)	25
				HY§	8,422	0	0	0		0	0	0	6
North	115,310	21 (0.18)	88	BJ	43,099	3	3	0		3 (0.07)	3 (0.07)	0	33
				TJ	42,320	7	5	2		6 (0.14)	5 (0.12)	1 (0.02)	32
				TS	29,891	23	7	16		12 (0.40)	4 (0.13)	8 (0.27)	23
East	127,474	28 (0.22)	97	NJ	70,153	16	4	12		14 (0.20)	4 (0.06)	10 (0.14)	53
				ZJ	57,321	14	12	2		14 (0.24)	12 (0.21)	2 (0.03)	44
Central	108,175	57 (0.53)	82	CS	40,521	19	15	4		19 (0.47)	15 (0.37)	4 (0.10)	31
				HB	67,654	38	17	21		38 (0.56)	17 (0.25)	21 (0.31)	51
South	157,949	76 (0.48)	120	GZ	66,477	74	18	56		36 (0.54)	12 (0.18)	24 (0.36)	51
				GD	40,628	36	24	12		31 (0.76)	23 (0.57)	8 (0.20)	31
				GX	50,844	13	10	3		9 (0.18)	8 (0.16)	1 (0.02)	39
West	84,535	1 (0.01)	64	NX§	8,795	2	1	1		0	0	0	7
				XJ	26,545	1	1	0		1 (0.04)	1 (0.04)	0	20
				XJ1§	8,777	2	0	2		0	0	0	7
				XJ2§	7,374	0	0	0		0	0	0	6
				CQ	33,044	0	0	0		0	0	0	25
*Values are no. (no./1,000 live births) except as indicated. BJ, Tsinghua University Hospital; CQ, Chongqing Health Center for Women and Children; CS, Changsha Hospital for Maternal and Child Health; EOD, early-onset disease; GBS, group B *Streptococcus*; GD, Guangdong Women and Children’s Hospital; GX, The Maternal and Child Health Hospital of Guangxi Zhuang Autonomous Region; GZ, Guangzhou Women and Children’s Medical Center; HB, Hubei Maternal and Child Health Hospital; HY, The First Affiliated Hospital, Harbin Medical University; LOD, late-onset disease; NE, northeast; NJ, Nanjing Maternity and Child Health Care Hospital; NX, General Hospital of Ningxia Medical University; SH, Children’s Hospital of Fudan University; SY, Shengjing Hospital, China Medical University; TJ, Tianjin Central Hospital of Gynecology Obstetrics; TS, Tangshan Maternal and Child Health Care Hospital; XJ, Maternal and Child Health Care Hospital of Uygur Autonomous Region; XJ1, The First Affiliated Hospital of Xi’an Jiaotong University; XJ2, The Second Affiliated Hospital of Xi’an Jiaotong University; ZJ, Women’s Hospital, Zhejiang University. †Estimated using highest incidence of 0.76/1,000 live births reported from this study. ‡Maternal and child hospitals, except where indicated. One hospital, Children’s Hospital of Fudan University, participated in the study but is omitted from this table because it does not have an obstetrics department, it had no infants with GBS born in it, and it was not included in calculation of incidence rates. §General hospital.													

**Figure 3:**
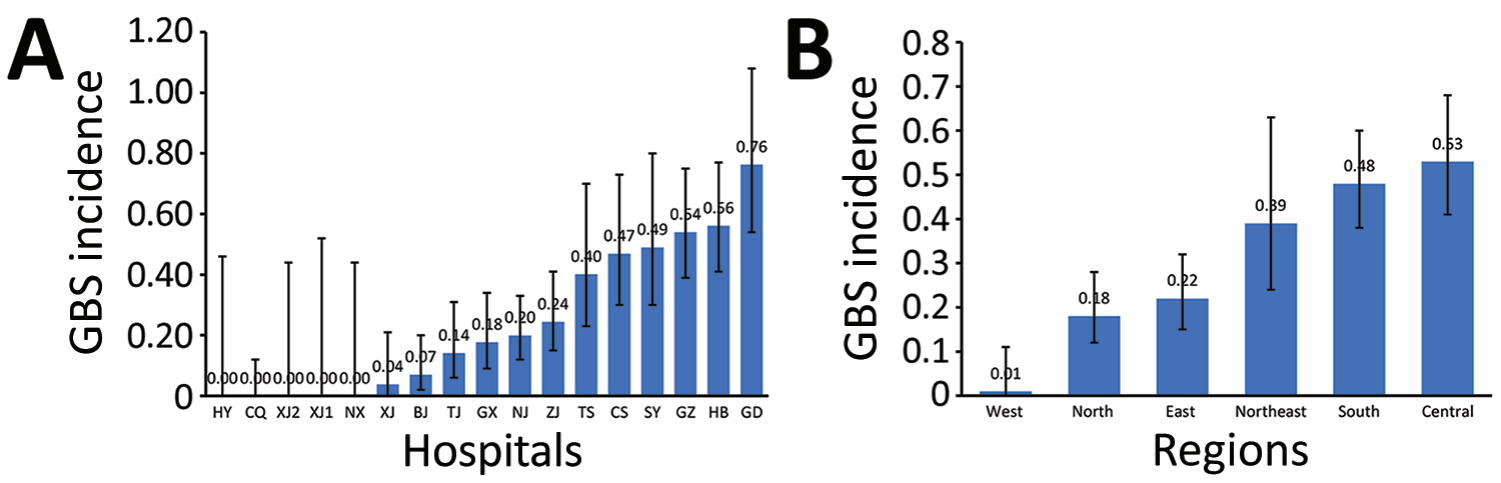
Incidence rate of invasive GBS disease (no. cases/1,000 live births) among infants <3 months of age, China, 2015–2017. A) Incidence by study hospital. B) Incidence by region. Error bars indicate 95% CIs. BJ, Tsinghua University Hospital; CQ, Chongqing Health Center for Women and Children; CS, Changsha Hospital for Maternal and Child Health; EOD, early-onset disease; GBS, invasive group B *Streptococcus*; GD, Guangdong Women and Children’s Hospital; GX, The Maternal and Child Health Hospital of Guangxi Zhuang Autonomous Region; GZ, Guangzhou Women and Children’s Medical Center; HB, Hubei Maternal and Child Health Hospital; HY, The First Affiliated Hospital, Harbin Medical University; LOD, late-onset disease; NJ, Nanjing Maternity and Child Health Care Hospital; NX, General Hospital of Ningxia Medical University; SH, Children’s Hospital of Fudan University; SY, Shengjing Hospital, China Medical University; TJ, Tianjin Central Hospital of Gynecology Obstetrics; TS, Tangshan Maternal and Child Health Care Hospital; XJ, Maternal and Child Health Care Hospital of Uygur Autonomous Region; XJ1, The First Affiliated Hospital of Xi’an Jiaotong University; XJ2, The Second Affiliated Hospital of Xi’an Jiaotong University; ZJ, Women’s Hospital, Zhejiang University

Guangdong Women and Children’s Hospital in the south reported the highest incidence rate, 0.76 (95% CI 0.54–1.08) cases/1,000 live births, along with Hubei Maternal and Child Health Hospital in the central region (0.56 [95% CI 0.41–0.77] cases/1,000 live births), and Guangzhou Women and Children’s Medical Center in the south (0.54 [95% CI 0.39–0.75] cases/1,000 live births) (Figure 3, panel A). Three participating hospitals reported no invasive GBS cases (Table 2). The incidence rate by region was highest in the central region (0.53 [95% CI 0.41–0.68] cases/1,000 live births), the south (0.48 [95% CI 0.38–0.60] cases/1,000 live births), and the northeast (0.39 [95% CI 0.24–0.63] cases/1,000 live births) and was lowest in the west (0.01 [95% CI 0–0.07] cases/1,000 live births) (Figure 3, panel B).

Our sensitivity analysis assumed an incidence rate of 0.76 cases/1,000 live births, the highest incidence rate reported by Guangdong Women and Children’s Hospital in our study (Table 2). Under this assumption, we estimated the annual number of cases of GBS in infants <3 months of age in China would be 13,604 among the country’s birth cohort of 17.9 million. Using the global case-fatality rate of 8.4%, we estimate China would have 1,142 GBS-associated deaths annually (*3*).

### Serotype Distribution

**Figure 4:**
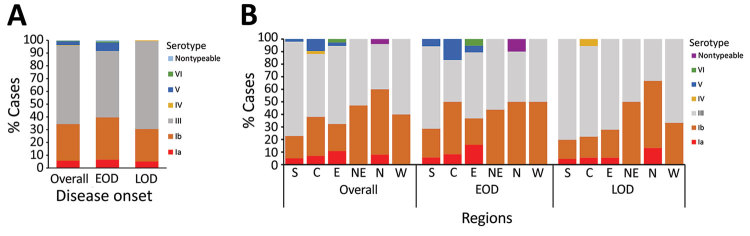
Distribution of invasive group B *Streptococcus* serotypes by disease onset (A) and region (B), China, 2015–2017. C, central; E, east; EOD, early-onset disease; LOD, late-onset disease; N, north; NE, northeast; S, south; W, west.

We typed 244 available isolates and identified 6 serotypes, Ia, Ib, III, IV, V, and VI. Serotype III (61.5%) was most pervasive, along with Ib (28.7%), and we saw fewer instances of Ia (5.7%) and V (2.9%), and 1 case (0.4%) each of serotypes IV and VI, as well as 1 nontypeable isolate (Figure 4, panel A). The relative distribution of serotypes differed between EOD and LOD (p = 0.004) (Figure 4, panel A); however, serotypes III and Ib were still most pervasive. Overall, the serotype distribution was similar across regions in China (Figure 4, panel B). The proportion of cases caused by serotype Ib was higher in the north and northeast than other regions (p = 0.002). Approximately two thirds of meningitis cases and 61.9% of sepsis cases were caused by serotype III, compared with 52.0% of pneumonia cases (p = 0.113). In contrast, 40% of diagnosed pneumonia was caused by serotype Ib compared with 30% of either sepsis or meningitis cases (p = 0.112).

### MLST Analysis

We performed MLST on all 244 serotyped isolates and identified 27 different sequence types (STs); 83.2% of which were ST17, ST19, ST10, ST12, and ST23. ST17 was most prevalent (89/244, 36.5%), along with ST19 (34/244, 13.9%), ST10 (33/244, 13.5%), and ST12 (32/244, 13.1%). Of ST17 isolates, 98% were serotype III, as were 88.2% of ST19 isolates; 2.2% of ST17 and 2.9% of ST19 were serotype Ib; and 8.8% of ST19 isolates were serotype V. Most ST10 (93.4%) and ST12 (93.8%) isolates were serotype Ib and 66.7% of ST23 isolates were serotype Ia.

**Figure 5:**
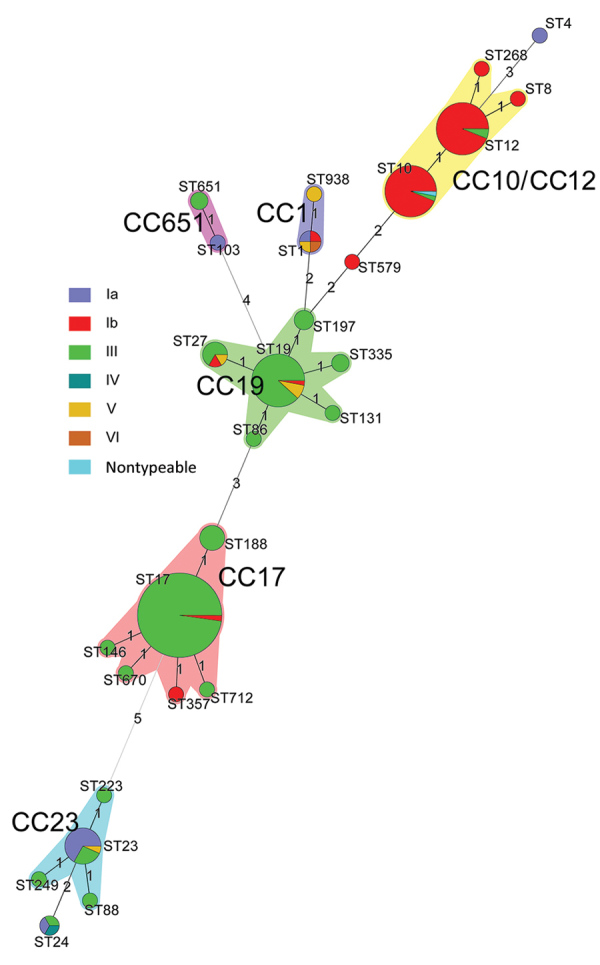
Minimum spanning tree of invasive group B *Streptococcus* (GBS) isolates by serotype showing the relationship between ST and clonal complex CC by serotype. Circles represent STs; size of each circle indicates the number of isolates within the specific type. The ST with the greatest number of single-locus variants is the founder ST. GBS isolate serotypes appear as different colors; shading denotes STs belonging to the same CC. CC, clonal complex; ST, sequence type.

**Figure 6:**
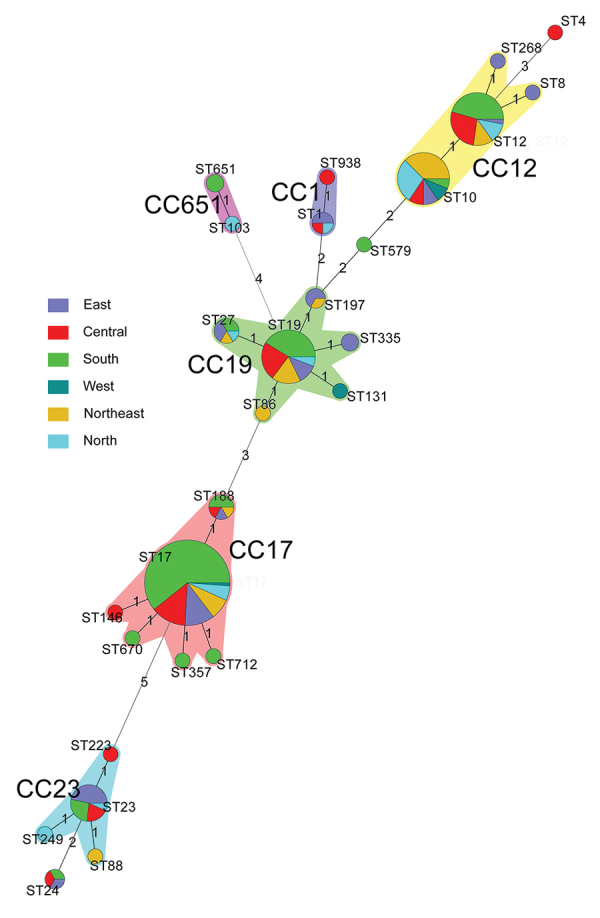
Minimum spanning tree analysis of invasive group B *Streptococcus* isolates showing the relationship between ST and CCs by region in China. Circles represent STs; size of each circle indicates the number of isolates within the specific type. The ST with the greatest number of single-locus variants is the founder ST. Regions appear as different colors; shading denotes STs belonging to the same CC. CC, clonal complex; ST, sequence type.

Twenty-seven STs clustered into 10 CCs (Figure 5). The most prevalent was CC17 (99/244 isolates, 40.6%), along with CC19 (47/244, 19.3%), CC12 (35/244, 14.3%), CC10 (32/244, 13.1%), and CC23 (18/244, 7.4%). Most CC17 and CC19 isolates were serotype III (137/150, 91.3%), and 90.0% of serotype Ib isolates were CC12 and CC10. CC17 and CC19 were dominant in the south, whereas CC10 and CC12 were common in the north and northeast (Figure 6). The proportion of CC17 isolates was higher in LOD (73/138, 52.9%) than in EOD (26/106, 24.5%) cases (p<0.0001). Strains belonging to CC19 and CC12 were less common in LOD than in EOD cases: CC19 in 14.5% (20/138) of LOD cases and 25.5% (27/106) of EOD cases (p = 0.031), and CC12 in 10.1% (14/138) of LOD and 19.8% (21/106) of EOD cases (p = 0.033).

## Discussion

GBS is associated with severe neonatal infection in some hospitals in China (*7*,*12*–*16*), but a paucity of generalizable data is available across the country. We conducted a large, multicenter study to evaluate the clinical and molecular epidemiology of invasive group B streptococcal disease among infants <3 months of age in China. We reported an overall GBS incidence rate of 0.31 (95% CI 0.27–0.36) cases/1,000 live births, which is lower than the estimated worldwide incidence rate of 0.49 (95% CI 0.43–0.56) cases/1,000 live births, but similar to estimates from elsewhere in Asia (0.30 [95% CI 0.43–0.56] cases/1,000 live births) (*3*). We estimated the incidence rates of EOD to be 0.18 cases/1,000 live births and LOD to be 0.13 cases/1,000 live births, which also are lower than estimated from a previous meta-analysis. That report estimated the global EOD incidence rate as 0.41 (95% CI 0.36–0.47) cases/1,000 live births and the LOD incidence rate as 0.26 (95% CI 0.21–0.30) cases/1,000 live births. The number of cases and birth cohort was one third more during the prospective than retrospective period, possibly because China implemented a 2-child policy in October 2015 (*17*). The incidence of invasive GBS was similar between the prospective and retrospective periods.

Despite implementing standard study and laboratory protocols at each participating hospital, we found substantial variability in the incidence rates between hospitals and regions in China. GBS incidence rates varied from 0 to 0.76 cases/1,000 live births at participating hospitals and from 0.01 to 0.53 cases/1,000 live births across regions. Similar geographic heterogeneity in incidence has been reported from South Africa, ranging from 0.03 to 2.72 cases/1,000 live births across 9 provinces (*18*). The authors of that study attributed the differences in incidence rates to biases in case detection, with varying thresholds for investigating and laboratory capacity for detecting GBS across the provinces, rather than actual differences (*18*). 

Elucidating the reasons for the regional differences in incidence of GBS in China could inform strategies hospitals might use to better identify such cases and improve care for patients with invasive GBS disease. We have several hypotheses for the differences in incidence rates we observed. Prior GBS research experience by staff in hospitals located in south and central China compared with those in other regions could have raised attending clinicians’ awareness of the need to investigate for GBS and given laboratory personnel more experience in microbiological identification of GBS. In addition, clinicians had discretion to conduct passive surveillance investigations, which could vary between personnel and study sites. Our study included only 5 general hospitals and 3 of these were located in the west, a region where we found a low GBS incidence rate. Typically, general hospitals have lower birth rates (Table 2), and some do not treat infants. For instance, the Maternal and Child Health Care Hospital of Uygur Autonomous Region has a policy to transfer all sick infants >28 days of age to the local children’s hospital for treatment. In addition, parents usually prefer to seek medical care for their infants at a children’s hospital rather than a general hospital. Therefore, our study might have missed some GBS cases born in general hospitals, resulting in lower incidence rates in these hospitals and the western region. Other explanations for regional differences include differences in maternal GBS colonization rates, strain virulence, challenges in accessing health services, and inadequate sterile sample collection capacity or improper methods. We will conduct further investigations on hospitals reporting no GBS cases without obvious reasons.

Our study identified a lower fatality rate (2.3%) than observed globally in a meta-analysis (8.4%; 95% CI 6.6%–10.2%), which could reflect early detection of suspected GBS and timely management with antimicrobial drugs. Also, we found that only 5.6% of surviving infants across all participating hospitals had neurologic sequelae at discharge. However, this value could be an underestimate because we did not use a standard assessment across sites and sequelae might not be obvious in infants. Further investigation in longer term follow-up studies in China are warranted to quantify the residual burden of neurologic sequelae in infants with invasive GBS disease.

Given that maternal GBS colonization is the primary risk factor for EOD, the US Centers for Disease Control and Prevention issued guidelines recommending intravenous intrapartum antibiotic prophylaxis (IAP) during labor to prevent EOD in infants born to women colonized with GBS or with risk factors (*19*). The IAP policy, along with universal screening, was shown to decrease EOD incidence but did not decrease LOD incidence (*19*). However, 37% of low- to middle-income countries, including China, have not implemented the IAP program on a systematic basis, and China currently does not have specific recommendations or guidelines for preventing GBS. During 2015–2017, eighteen hospitals in this study reported only 13.2% of GBS case-mothers received GBS screening before delivery. Currently, not every hospital has a GBS screening program. Some hospitals offer optional GBS screening, but patients must cover the cost, and screening is not covered by any medical insurance. We also have seen previously that surveyed pregnant women thought GBS incidence was low and most chose not to have GBS screening (W. Ji, H. Liu, and Y. Fang, unpub. data).

Vaccination of pregnant women against GBS is a promising strategy to prevent invasive GBS disease in their infants (*20*). The World Health Organization proposed priority research and development pathways for GBS vaccines to facilitate and accelerate vaccine licensure (*21*). Our results indicated that serotype III was the most common type (61.2%). Other studies also have reported the comparable proportion of serotype III in China (*7*,*22*–*24*). In general, GBS serotype distributions were similar across regions of China, and were similar to findings for other countries, with serotype III dominating (61.5%) and serotypes Ia, Ib, II, III, and V causing 97% of invasive GBS disease (*3*). However, serotype Ib accounted for a higher proportion of disease in the northeast and north in China compared with other regions. The 244 isolates belonged to 27 STs and were clustered into 5 main CCs. ST17/CC17 and ST19/CC19 were found almost exclusively in serotype III, which is comparable with findings from other studies (*12*,*25*–*27*). The ST17 clone of serotype III is known to be a hypervirulent strain and a leading cause of meningitis in neonates (*28*,*29*). A pentavalent GBS vaccine including serotypes Ia, Ib, II, III, and V (*30*) or a hexavalent vaccine including serotypes Ia, Ib, II, III, IV, and V would cover all invasive GBS disease–causing serotypes in China.

Our study has several limitations. First, all participating hospitals were large urban tertiary hospitals. We did not include secondary hospitals and primary care institutions mainly because they lacked adequate laboratory facilities for GBS culture and identification of invasive GBS disease, which might have led to delayed confirmation of EOD cases. We found 56.2% of EOD cases were identified <24 hours after birth. Findings might differ in primary and secondary settings because of differences in populations and clinical practice, so these settings should be included in future studies. Second, the retrospective collection of data during January 2015–May 2016 did not include all isolates and only 46.3% were available for strain analysis. Last, rates of GBS could be underestimated because our study did not account for GBS in infants treated at nonstudy hospitals after discharge from study hospitals.

In summary, our large generalizable study on invasive GBS disease in infants in China informs the current clinical and molecular epidemiology of GBS. Despite the low rates of fatality and detectable neurologic sequelae at discharge, clinicians, laboratory technicians, and epidemiologists should be aware that GBS is prevalent, especially in the south and central regions of China. Future work and continuous surveillance are warranted to better understand the heterogeneity of incidence across regions, identify risk factors, and conduct long-term follow-up to further assess the prevalence of invasive GBS disease on neurologic development in surviving infants.

Appendix Additional information on invasive group B *Streptococcus* infections in infants <3 months of age, China.
