# Supplementary material for: Clinical and Molecular Epidemiology of Invasive Group B Streptococcus Disease among Infants, China
Source: Emerg Infect Dis. 2019 Nov;25(11):2021–30. doi: 10.3201/eid2511.181647 (PMC6810193; doi:10.3201/eid2511.181647)
Supplement: Appendix — Additional information on invasive group B Streptococcus infections in infants <3 months of age, China. [file 18-1647-Techapp-s1.pdf]

Article DOI: https://doi.org/10.3201/eid2511.181647

Clinical and Molecular Epidemiology of Invasive Group B *Streptococcus* Disease among Infants, China

Appendix

Appendix Table 1. Characteristics of infants <3 months of age with invasive group B *Streptococcus* infection, by study period and disease onset, China, 2015–2017*

	Retrospective period			Prospective period			
Characteristics	EOD (n = 64)	LOD (n = 59)	Total (n = 123)	EOD (n = 82)	LOD (n = 99)	Total (n = 181)	p value
Median case age at GBS diagnosis, d (IQR)	1 (0–2)	21 (17–40)	6 (1–21)	0 (0–1)	23 (14–51)	6 (1–21)	0.018
Gender, no. (%)							
M	32 (50.0)	27 (45.8)	59 (48.0)	42 (51.2)	37 (37.4)	79 (43.6)	0.458
F	32 (50.0)	32 (54.2)	64 (52.0)	40 (48.8)	62 (62.6)	102 (56.4)	
Median birth weight, g (IQR)	2,972 (2,470–3,350)	3,100 (2,500–3,450)	3,005 (2,500–3,400)	3,260 (2,900–3,520)	3,280 (2,850–3,500)	3,005 (2,500–3,400)	0.001
1,500–2,500; no. (%)	13 (20.3)	12 (20.3)	25 (20.3)	13 (15.9)	9 (9.1)	22 (12.2)	0.076
<1,500; no. (%)	4 (6.3)	3 (5.1)	7 (5.7)	1 (1.2)	5 (5.1)	6 (3.3)	
Delivery hospital, no. (%)							
Sentinel	51 (79.7)	28 (47.5)	79 (64.2)	64 (78.1)	56 (56.6)	120 (66.3)	0.709
Other	13 (20.1)	31 (52.5)	44 (35.8)	18 (21.9)	43 (43.4)	61 (33.7)	
Median gestational age, wk (IQR)	39 (36–40)	39 (37–40)	39 (37–40)	39 (37–40)	39 (37–40)	39 (36–40)	0.209
Term, no. (%)							
<34 wk	10 (15.6)	6 (10.2)	16 (13.0)	7 (8.5)	7 (7.1)	14 (7.7)	0.252
34–<37 wk	9 (14.1)	9 (15.3)	18 (14.6)	13 (15.9)	10 (10.1)	23 (12.7)	
≥37 wk	45 (70.3)	44 (74.6)	89 (72.4)	62 (75.6)	82 (82.8)	144 (79.6)	
Delivery type, no. (%)							
Vaginal	40 (62.5)	41 (69.5)	81 (65.9)	57 (69.5)	53 (53.5)	110 (60.8)	0.587
C-section	20 (31.3)	17 (28.8)	37 (30.1)	20 (24.4)	45 (45.5)	65 (35.9)	
Forceps	3 (4.7)	0	3 (2.4)	4 (4.9)	1 (1.0)	5 (2.8)	
Unknown	1 (1.6)	1 (1.7)	2 (1.6)	1 (1.2)	0	1 (0.6)	
Bacterial infections, no. (%)†							
Sepsis	55 (85.9)	45 (76.3)	100 (81.3)	67 (81.7)	76 (76.8)	143 (79.0)	0.624
Pneumonia	31 (48.4)	16 (27.1)	47 (38.2)	45 (54.9)	34 (34.3)	79 (43.7)	0.345
Meningitis	8 (12.5)	28 (47.5)	36 (29.3)	10 (12.2)	41 (41.4)	51 (28.2)	0.836
Sepsis and pneumonia	24 (37.5)	14 (23.7)	38 (30.9)	38 (46.3)	21 (21.2)	59 (32.6)	0.755
Sepsis and meningitis	5 (7.8)	15 (25.4)	20 (16.3)	2 (2.4)	28 (28.3)	30 (16.6)	0.714
Pneumonia and meningitis	0	0	0	2 (2.4)	2 (3.0)	4 (2.2)	0.240
Clinical symptoms, no. (%)†							
Fever	22 (34.4)	42 (71.2)	64 (52.0)	16 (19.5)	79 (79.8)	95 (52.5)	0.996
Breathing problems	26 (40.6)	11 (18.6)	37 (30.1)	57 (69.5)	27 (27.3)	84 (46.4)	0.005
Cyanosis	13 (20.3)	4 (6.8)	17 (13.8)	19 (23.2)	6 (6.1)	25 (13.8)	0.976
Seizures	1 (1.6)	5 (8.5)	6 (4.9)	3 (3.7)	15 (15.2)	18 (9.9)	0.112
Limpness or stiffness	3 (4.7)	0	3 (2.5)	2 (2.4)	10 (10.1)	12 (6.6)	0.101
Poor feeding	4 (6.3)	7 (11.9)	11 (9.0)	9 (11.0)	20 (20.2)	29 (16.0)	0.077
Irritability	3 (4.7)	3 (5.2)	6 (4.9)	1 (1.2)	12 (12.1)	13 (7.2)	0.425

Characteristics	Retrospective period EOD (n = 64)	Retrospective period LOD (n = 59)	Retrospective period Total (n = 123)	Prospective period EOD (n = 82)	Prospective period LOD (n = 99)	Prospective period Total (n = 181)	p value
Median length of hospitalization, d (IQR)	15 (12–21)	22 (13–39)	16 (12–27)	15 (12–19)	17 (13–30)	16 (12–27)	0.073
Outcome, no. (%)							
Recovered	43 (67.2)	35 (59.3)	78 (63.4)	62 (75.6)	65 (65.7)	127 (70.2)	0.578
Transferred to other hospitals	3 (4.7)	4 (6.8)	7 (5.7)	1 (1.2)	9 (9.1)	10 (5.5)	
Died	1 (1.6)	3 (5.1)	4 (3.3)	3 (3.7)	0	3 (1.7)	
Abnormal neurology at discharge	1 (1.6)	9 (15.3)	10 (8.1)	4 (4.9)	3 (3.0)	7 (3.9)	
Condition improved	7 (10.1)	4 (6.8)	11 (8.9)	5 (6.1)	12 (12.1)	17 (9.4)	
Discharge requested	9 (14.1)	4 (6.8)	13 (10.6)	7 (8.5)	10 (10.1)	17 (9.4)	

*Prospective period, May 5, 2016–December 31, 2017; retrospective period, January 1, 2015–May 4, 2016. EOD, early-onset disease; GBS, invasive group B *Streptococcus*; LOD, late-onset disease; SD, standard deviation; IQR, interquartile range.
†Cases could have >1.

Appendix Table 2. Incidence of invasive group B streptococcal disease among infants ≤3 months of age by region, China, 2015–2017*

Region	GBS incidence/1,000 live births (95% CI) Retrospective period	GBS incidence/1,000 live births (95% CI) Prospective period
West	0 (0–0.12)	0.02 (0–0.11)
North	0.21 (0.11–0.40)	0.17 (0.10–0.29)
East	0.16 (0.08–0.31)	0.26 (0.17–0.40)
Northeast	0.36 (0.17–0.79)	0.41 (0.22–0.75)
South	0.50 (0.35–0.70)	0.47 (0.35–0.63)
Central	0.59 (0.40–0.88)	0.48 (0.34–0.68)
Total	0.32 (0.26–0.40)	0.31 (0.26–0.37)

*Prospective period, May 5, 2016–December 31, 2017; retrospective period, January 1, 2015–May 4, 2016. GBS, invasive group B *Streptococcus*.

Appendix Table 3. Characteristics of case-fatalities due to invasive group B streptococcal disease among infants ≤3 months of age, China, 2015–2017*

Case no.	Region	Birth hospital†	Sex/ gestation, wk	Birth wt, g	Clinical manifestation	Disease onset	Delivery type	No. days from admission to death	Serotype	ST
Prospective period										
CS07	C	Sentinel	F/39	3,050	Pneumonia	EOD	Vaginal	1	Ib	12
GX01	S	Sentinel	F/40	3,380	Sepsis and pneumonia	EOD	Vaginal	5	Ib	12
SY04	NE	Other	F/32	2,050	Sepsis and shock	EOD	C-section	3	Ib	12
Retrospective period										
GX11R	S	Other	M/39	2,600	Pneumonia	EOD	Unknown	2	NA	NA
GZR07	S	Sentinel	F/39	3,200	Sepsis and meningitis	LOD	Vaginal	6	III	17
GZR30	S	Other	M/38	3,360	Sepsis, pneumonia, and shock	LOD	Vaginal	29	III	17
SYR09	NE	Sentinel	F/30	1,213	Sepsis, meningitis, and shock	LOD	C-section	2	Ib	10

*Prospective cases occurred during May 5, 2016–December 31, 2017; retrospective cases occurred during January 1, 2015–May 4, 2016. C, central; EOD, early onset disease; LOD, late onset disease; NE, northeast; S, south.
